# Public Perception of COVID-19 Vaccination in Italy: The Role of Trust and Experts’ Communication

**DOI:** 10.3389/ijph.2022.1604222

**Published:** 2022-03-16

**Authors:** Massimiano Bucchi, Eliana Fattorini, Barbara Saracino

**Affiliations:** ^1^ Department of Sociology and Social Research, University of Trento, Trento, Italy; ^2^ Department of Political and Social Sciences, University of Bologna, Bologna, Italy

**Keywords:** trust, COVID-19 vaccine, science communication, experts, science and society, vaccine acceptance

## Abstract

**Objectives:** Vaccination campaigns against COVID-19 throughout the world are not only a major organisational challenge, but also a communication and social challenge. Recent data from several countries show that a relevant proportion of citizens either do not plan to be vaccinated against COVID-19 or would rather postpone their vaccination. We argue that such attitudes are not the result of generalised scepticism about vaccination, nor of generalised distrust in science.

**Methods:** We analysed data from three survey waves on attitudes to vaccination against COVID-19 conducted in Italy in October 2020, January 2021, and May 2021 in the context of the Science in Society Monitor.

**Results:** Positive evaluations of experts’ communication and trust in their contribution—as well as in that of health institutions, local authorities, and healthcare workers—play a key role in understanding the willingness to be vaccinated.

**Conclusion:** Relevant implications can be drawn in terms of communication efforts and institutional strategies that are essential to build effective and inclusive vaccination campaigns.

## Introduction

The COVID-19 pandemic is not only a health, economic and social crisis but also represents an extraordinary opportunity for studying public perception of the role of science and scientific experts in emergencies [1–4]. The COVID-19 pandemic has been characterised by an unprecedented level of involvement of scientific experts in media coverage and public exposure to experts’ advice. In this paper we analysed the role of informative sources, with a particular focus on the relationship between perceptions and evaluations of the communicative role of scientific experts and attitudes towards anti-COVID-19 vaccines.

Since Goodell’s seminal work on visible scientists [5], the role of scientific experts in public communication has become increasingly important due to changes in the media landscape as well as in the dynamics between science and society [6–9]. In the past decades, social media have provided a platform for experts to engage more actively and directly in public debate [10, 11]. Discussions and controversies among experts previously confined to specialist communication contexts have become—at least potentially—accessible to broader audiences [12–14]. From the beginning of the COVID-19 emergency scientific communication has played a key role both in framing the issue and in providing citizens with relevant information and instructions on how to minimize the risk of contagion. In Italy as in other countries, information has been provided from a variety of national and international actors—i.e., World Health Organization, government, Civic Protection, National Institutes of Health, local authorities, healthcare workers. Moreover, information and health procedures have been communicated through a variety of media: radio, television, newspapers, institutional websites, and social media. Unlike other countries [15], however, in Italy the mediatisation of science [16–18] has started since the beginning of the pandemic: the Italian media have given a daily visibility to a variety of experts—virologists, epidemiologists, but also physicists and data scientists—often providing different and sometimes even contradictory views on the evolution and management of the pandemic.

As pointed out by Peters et al. (6), among the key elements that the experts must deal with when speaking publicly are communicative clarity and trust.

A widespread concern that has characterised the COVID-19 pandemic—in addition to health, economic, psychological, and political ones—is the so called “infodemic.” This term mainly refers to the diffusion of inaccurate information—also known as “fake news”—especially through social media [19, 20]. This characterisation has often been linked to a representation of passive and uncritical audiences: the assumption is that people have searched for information about the Coronavirus mainly on social media and, therefore, have been easily misguided by this information. Outcry for raging mistrust of science and of scientific expertise is also often associated with this description of audiences, although the causal connection is not always clearly specified—i.e., whether such mistrust is a product of, or rather contributes to enhance, the circulation of inaccurate information [21–23]. We tested these arguments using empirical data on public perception during different stages of the pandemic in Italy.

Although Italy has now a high percentage of population vaccinated against COVID-19, during the past year a relevant proportion of citizens expressed the intention not to be vaccinated against COVID-19 or to postpone their vaccination [24]. Vaccine hesitancy can be defined as a “delay in acceptance or refusal of vaccination despite availability of vaccination services” [25]. According to Eurobarometer [26], in 2019 25% of Italian citizens tended to or totally disagree on the importance of routine vaccination, and this value is among the highest levels of vaccine hesitancy recorded in the European countries after France (30%). These hesitant attitudes cannot be explained on the basis of generalised scepticism about vaccination or of generalised distrust in science and scientists. Actually, trust in science has been constantly high during the past few years in Italy, with an additional increase during the pandemic recorded in Italy as well as in other European countries [27–29].

Using data from three surveys waves conducted in Italy in October 2020, January 2021, and May 2021 in the context of the Science in Society Monitor with a specific focus on COVID-19 vaccination, we decided to test two hypotheses:

H1 The higher the trust in scientific experts, healthcare workers, and international health organizations, the higher the willingness to be vaccinated.

H2 The more positive the evaluation of experts’ communication, the higher the willingness to be vaccinated quickly.

## Methods

We analyzed data of three waves of a survey conducted in October 2020, in January 2021, and in May 2021 in the context of the Science in Society Monitor.^1^ Data were collected through CATI (Computer Assisted Telephone Interview) and CAWI (Computer Assisted Web) interviews—respectively for 30% and 70% of the sample—at a national level. The sample is proportional and representative by gender, age, and area of residence of Italian population aged over 15 years.

More specifically, the first wave of the survey was conducted between 21 and 30 October 2020 interviewing 1,001 subjects; total cases became 991 after weighing^2^ to make the sample structure proportional to the Italian population regarding gender, age, and educational level. The second wave was conducted between 20 and 25 January 2021 interviewing 1,007 subjects, 987 after weighing. The third wave was conducted between 17 and 28 May 2021 interviewing 1,003 subjects, 977 after weighing. In particular, all the considered waves collected data on public perceptions about: trust in national scientific experts and other key actors (e.g., health institutions, political administrators, media); evaluations of experts’ public communication during the Covid-19 pandemic; use and information sources and judgement of their trustworthiness.

In the next section, after analyzing monovariate distributions of the responses collected in October 2020, January 2021, and May 2021, we will present the results of a binomial multivariate logistic regressions performed for each wave.^3^

As dependent variable we selected the question related to the willingness to be vaccinated that has three response categories in October 2020 (“be vaccinated as soon as possible,” “be vaccinated but not immediately,” and “do not plan to be vaccinated”). In Italy the vaccination campaign started in late December 2020; to account for this, in January 2021 and May 2021 the answer option “already vaccinated” was added to the questionnaire. As of January, roughly 1% of respondents is vaccinated; while in May 2021 there is a significant increase in the number of people already vaccinated (45%). For this reason, the categories “be vaccinated as soon as possible” and “already vaccinated” were later aggregated. We will refer from here on only to the category “get vaccinated as soon as possible.”

Independent variables included in the analysis are: gender; age group; level of education; main source of information; the most trusted source of information; evaluation of national scientific experts’ public communication; perception of the pandemic threat severity; trust in key actors (general practitioners and healthcare workers, scientists, national health institutions, international health institutions, national political administrators, local political administrators, journalists).

In October 2020, the variable related to trust was specifically oriented to identify public trust in the ability of scientists in finding a solution to the pandemic. This topic was then further explored in the January and May 2021 waves, in which this item was replaced by a battery of items related to trust in key actors (including scientists).

## Results

In all three waves, the sources of information most widely consulted by Italians during the pandemic are television and/or radio news programs. Specifically, in October 2020 54% of Italians rely on these sources when searching for COVID-19 news; 52% of respondents in January 2021 and 29% in May 2021 rely on television and/or radio news programs when searching for information about the anti-COVID-19 vaccines. Indeed, in May 2021 this decrease resulted in 26% of respondents who consult institutional sources of information about the anti-COVID-19 vaccines—with an increase of almost 10% points compared to January 2021—and 20% on their own general practitioner—increased by nearly 12 points. Health precaution information provided by health institutions and political (local and national) authorities are the most trusted in October 2020 (43%). 37% of respondents in January 2021 and nearly 40% in May 2021 rely on these institutional sources when searching for information about anti-COVID-19 vaccines. It should be noted that in all three waves, respondents express very little trust in social media or web forums when searching for information about the health precautions to be taken against COVID-19 or anti-COVID-19 vaccine-related information—only 4% of subjects rely on this source of information.

The study shows trust in science to be very high. In May 2021, scientists are perceived the most trustworthy key actors during the pandemic (90%)—and even in January 2021 scientists were among the most trusted (89%). Follow physicians and healthcare workers (88%)—91% in January 2021—and health national authorities (74%)—77% in January 2021.

However, much less positive is the judgement of scientific experts’ public communication performances. Respondents were asked to the evaluate public statements and comments on TV/radio, daily press, social media by scientific experts about the pandemic: more than half of the respondents in all three waves perceive those speeches as mostly confusing—62% in October 2020, 60% in January 2021, and 63% in May 2021. Indeed, in May 2021 only nearly 15% of respondents consider national experts’ public communication clear—with a slight decrease of 5 percentage points since January 2021.

Regarding attitudes towards vaccines, in October 2020—the COVID-19 vaccination campaign in Italy started in late December 2020—only 36% of Italians express their willingness to get vaccinated as soon as possible. In January 2021 the proportion of people that are already vaccinated or that will be vaccinated as soon as possible has increased reaching 60%. In May 2021, nearly 84% of respondents express their willingness to be vaccinated or have been already vaccinated.^4^

Further insights come from looking at the specific reasons behind this pro-vaccine attitude: in May 2021 more than a half of Italians believe that vaccines benefits exceed risks (52%)—with an increase of 17 percentage points compared to January 2021—and 21% trust scientists—with a decrease of 16 percentage points. In both January 2021 and May 2021 waves, the main motivation of those who are not planning to be vaccinated is the hastily approval procedure of anti-COVID-19 vaccines—respectively, 45% and 37%. In May 2021 the percentage of respondents who are against vaccines in general is 28%—with an increase of 12 percentage points since January. Looking at the motivations behind these specific vaccine hesitant attitudes, the percentage of respondents who do not trust pharmaceutical companies increased by 6 percentage points from January 2021 (11%) to May 2021 (17%). The less cited reasons to refuse vaccination are worrying news about anti-COVID-19 vaccines—19% in January 2021 and 4% in May 2021—and suffering from serious illnesses/allergies—10% in January 2021 and 11% in May 2021 (Table 1).

**TABLE 1:** Monovariate distributions of the responses collected (%) (Observa Science in Society Monitor, Italy. October 2020, January 2021, May 2021).

	October 2020 (*n* = 991)	January 2021 (*n* = 987)	May 2021 (*n* = 977)
Where are you mainly looking for information about COVID-19?			
TV/Radio news	54.3	51.5	28.9
Daily press	15.6	12.2	10.2
Institutional web sources (e.g., Health Ministry, Region)	18.8	16,3	26
Social media contacts	3.8	2,5	2
My own GP	3.4	8,3	20
Relatives or friends	—	1.4	4.2
Other	1.7	1.8	3.2
I am not looking for information	2.4	6	5.5
Regarding precautions to avoid contagion. Which of these sources do you trust most?			
Indications from national and local institutions	42.9	36.8	39.5
My own GP	23.8	33.4	35.3
Newspaper articles	3.3	4.3	4.4
TV/Radio broadcasts	18.1	16.1	11.3
Relatives or friends	4.2	5.4	5.5
Pharmacists	3.6	—	—
Web forums or social media contacts	4.1	4	4
Thinking about the Italian scientific experts who have intervened publicly on the pandemic. Which of these statements best refelcts your thinking?			
Public interventions of Italian scientific experts have been clear and effective	19.2	18.9	14.7
The opinions of Italian scientific experts have been too disparate and caused confusion	61.9	59.5	62.9
Most scientific experts are competent but unable to communicate clearly	9.7	11.2	11.2
It would be better if scientific experts gave their opinions to institutions confidentially and not publicly	9.2	10.4	11.2
Thinking about science and scientists. you believe that			
Scientists will soon find solutions to eliminate the risk	19.8		
Scientists will find solutions. but the timeframe will be rather long	70.6		
I do not think scientists will be able to find a solution	9.6		
Overall, you consider this pandemic			
A serious and concrete threat, against which many precautions are necessary to defend oneself	60.3	69.8	
A real risk, but overestimated by politics and media	29.8	26.7	
A total invention to justify political and economic decisions	6.5	3	
DK	3.4	0.5	
When a vaccine will be available, will you personally			
Get vaccinated as soon as possible	36.2	58.7	38.5
Get vaccinated, but not immediately	37.9	23.9	10
I do not plan to get vaccinated	21.5	14.3	6
I have already been vaccinated	—	1.2	45.3
DK	4.4	1.9	0.2
How much trust do you personally place in Physicians and health care workers?			
A lot or enough		91	87.9
Little or not at all		8.9	10.9
DK		0.1	1.2
How much trust do you personally place in Scientists?			
A lot or enough		88.5	89.8
Little or not at all		9.8	9.1
DK		1.7	1.1
How much trust do you personally place in National health authorities (e.g., ISS and AIFA)?			
A lot or enough		77.2	73.7
Little or not at all		22.3	23.9
DK		0.5	2.4
How much trust do you personally place in International health authorities (e.g., WHO or EMA)?			
A lot or enough		73.4	67
Little or not at all		23.8	28.5
DK		2.8	4.5
How much trust do you personally place in National political administrators?			
A lot or enough		44.5	30.6
Little or not at all		54.2	67.2
DK		1.3	2.2
How much trust do you personally place in Local political administrators?			
A lot or enough		33.0	36.6
Little or not at all		64.3	61.7
DK		2.7	1.7
How much trust do you personally place in Journalists?			
A lot or enough		31.9	29.5
Little or not at all		67.1	69.5
DK		1	1

The results of the binomial multivariate logistic regressions show that in October 2020, male citizens are nearly 59% more likely than females to get vaccinated as soon as possible. Age also has a positive influence on increasing the likelihood to be vaccinated against COVID-19: people aged 60+ are 74% more likely to be vaccinated as soon as possible compared to respondents aged 15–29. Moreover, people with a high or medium level of education are more likely—respectively by 56% and 67%—to be positive about receiving the vaccine. The binomial multivariate logistic regressions for January 2021 show that males are 94% more likely to reply that they would get vaccinated as soon as possible. For the same dependent variable, compared to people aged 15–29, respondents aged 60+ are significantly more likely to be already vaccinated or to be vaccinated as soon as possible. Regarding the educational level, the trend highlighted in October 2020 is confirmed also in January 2021: net of the effects of control variables, the higher the level of education, the higher the likelihood to accept the anti-COVID-19 vaccine. Indeed, compared to those with a low level of education, citizens with a high level of education are considerably more likely to accept to be immunized against COVID-19. Analyzing the binomial multivariate logistic regression for May 2021, as in October 2020 and January 2021 models, age has a role in positively influencing the likelihood to be vaccinated. Indeed, respondents aged 60+ are significantly more likely to be vaccinated as soon as possible compared to the younger cohorts. Instead, gender and level of education cease to be statistically significant.

With regards to the perception of the pandemic threat in October 2020, controlling for other control variables, Italians who agree with the statement that COVID-19 health emergency is “a serious and real threat from which many precautions are necessary to protect against” [odds ratio (OR) = 3.331; 95% confidence interval (CI): 1.463, 7.580] are significantly more likely to be willing to receive the vaccine as soon as possible compared to subjects who consider the pandemic “a fabrication to justify political and economic decisions.” Similarly, in January 2021 respondents who recognize the COVID-19 pandemic as a real threat are considerably more likely (OR = 3.297; 95% CI: 1.041, 10.439) to be vaccinated as soon as possible than those who think that the pandemic is a political and economic fabrication.

The source of information can influence the willingness to be vaccinated against COVID-19. Interestingly, in January 2021 Italians who rely on their own family and friends as main source of information are 83% less likely to be vaccinated as soon as possible compared to those who search for information mainly on TV or radio news programs. In addition, in May 2021, citizens who search for information mainly on daily newspapers (OR = 3.961; 95% CI: 1.675, 9.371) or on institutional websites (OR = 5.359; 95% CI: 1.957, 14.675) are more likely to be vaccinated.

Positive evaluations of experts’ communication and trust in their contribution—as well as in that of national health and political institutions—are particularly important to understand attitudes towards anti-COVID-19 vaccines. For example, in October 2020 trust in the ability of scientists to find solutions in the short term to deal with the pandemic is positively associated with the likelihood to get vaccinated. Particularly, compared to Italians who do not trust scientists, citizens who believe that scientists will find a solution quickly are more likely (OR = 3.141; 95% CI: 1.441, 6.847) to get vaccinated as soon as possible. In January 2021, the positive evaluation of national scientific experts’ communication during the pandemic increases the likelihood to be vaccinated as soon as possible. Compared to citizens who think that national scientific experts’ opinions should have been communicated confidentially to institutional authorities rather than publicly, Italians who consider their communication clear and effective are more likely (OR = 2.199; 95% CI: 1.050, 4.607) to be vaccinated as soon as the vaccine will become available. This is confirmed even in May 2021 model: Italians who consider experts’ communication confusing are nearly 72% less likely to be vaccinated. Moreover, trust in key actors seems to play an important role in the willingness to get vaccinated. Specifically, net of the effects of other variables, in January 2021 people who trust healthcare workers (OR = 3.043; 95% CI: 1.323, 6.997), scientists (OR = 2.150; 95% CI: 1.060, 4.362), national health institutions (OR = 2.497; 95% CI: 1.476, 4.225), and local authorities (OR = 2.394; 95% CI: 1.569, 3.650) are more likely to accept the anti-COVID-19 vaccines. Even in May 2021, trust in healthcare workers (OR = 2.326; 95% CI: 1.164, 4.648), national health institutions (OR = 2.046; 95% CI: 1.116, 3.749), and local authorities (OR = 2.015; 95% CI: 1.145, 3.544) positively influences the likelihood to be vaccinated (Tables 2–4).

**TABLE 2:** Effects on the willingness to be vaccinated as soon as possible: binomial multivariate logistic regression on the data collected in October 2020 (*n* = 991) (Observa Science in Society Monitor, Italy. October 2020).

		Sig.	OR	95% CIs
Gender	Male vs. Female	**	1.586	1.170–2.151
Age group	15–29 years (ref.)	***		
	30–44 years		0.701	0.439–1.121
	45–59 years		0.747	0.476–1.173
	60 years and over	*	1.742	1.077–2.820
Level of education	Low (ref.)	*		
	Medium	*	1.557	1.099–2.207
	High	*	1.677	1.082–2.600
Main source of information	TV/Radio news (ref.)			
	Daily press		1.066	0.698–1.630
	Institutional web sources		0.835	0.555–1.256
	Social media contacts		0.883	0.346–2.250
	Own GP		1.265	0.564–2.835
	Other		0.797	0.247–2.569
	Not looking for information		0.293	0.071–1.213
The most trusted source of information	Indications from national and local institutions (ref.)	**		
	Own GP		1.191	0.799–1.776
	Newspaper articles	**	2.956	1.317–6.637
	TV/Radio broadcasts		0.682	0.435–1.069
	Relatives or friends		1.573	0.660–3.751
	Pharmacists		0.844	0.375–1.902
	Web forums or social media contacts	*	2.203	1.002–4.846
Evaluation of the national scientific experts’ public communication	Opinions to institutions confidentially and not publicly (ref.)	*		
	Clear and effective interventions		1.369	0.747–2.510
	Too disparate opinions and cause of confusion		0.766	0.454–1.294
	Experts unable to communicate clearly		0.857	0.420–1.747
Perceptions of the pandemic	A total invention (ref.)	***		
	A serious and concrete threat	**	3.331	1.463–7.580
	A real risk		1.154	0.502–2.653
Trust in the ability of scientists in finding solutions	Never (ref.)	**		
	Soon	**	3.141	1.441–6.847
	Not soon		1.880	0.920–3.842
	Costant	***	0.090	

Significance: *p < 0.05, **p < 0.01, ***p < 0.001.

**TABLE 3:** Effects on the willingness to be vaccinated as soon as possible: binomial multivariate logistic regression on the data collected in January 2021 (*n* = 987) (Observa Science in Society Monitor, Italy. January 2021).

		Sig.	OR	95% CIs
Gender	Male vs. Female	***	1.939	1.349–2.786
Age group	15–29 years (ref.)	***		
	30–44 years	**	0.440	0.258–0.750
	45–59 years	*	0.560	0.334–0.939
	60 years and over	***	2.757	1.505–5.050
Level of education	Low (ref.)	**		
	Medium		1.249	0.834–1.870
	High	***	2.649	1.532–4.580
Main source of information	TV/Radio news (ref.)	**		
	Daily press		0.734	0.429–1.259
	Institutional web sources		1.615	0.972–2.684
	Social media contacts		1.257	0.462–3.421
	Own GP		0.609	0.326–1.138
	Relatives or friends	*	0.166	0.032–0.860
	Other		3.864	0.857–17.412
	Not looking for information		1.035	0.441–2.433
The most trusted source of information	Indications from national and local institutions (ref.)	**		
	Own GP		0.992	0.644–1.527
	Newspaper articles		0.525	0.228–1.208
	TV/Radio broadcasts		0.721	0.419–1.241
	Relatives or friends	**	4.048	1.572–10.427
	Web forums or social media contacts		0.508	0.180–1.433
Evaluation of the national scientific experts’ public comm	Opinions to institutions confidentially and not publicly (ref.)			
	Clear and effective interventions	*	2.199	1.050–4.607
	Too disparate opinions and cause of confusion		1.286	0.673–2.459
	Experts unable to communicate clearly		1.036	0.469–2.286
Perceptions of the pandemic	A total invention (ref.)	***		
	A serious and concrete threat	*	3.297	1.041–10.439
	A real risk		0.832	0.258–2.686
Trust in physicians and health care workers (A lot or enough vs. Little or not at all)		**	3.043	1.323–6.997
Trust in scientists (A lot or enough vs. Little or not at all)		*	2.150	1.060–4.362
Trust in national health institutions (A lot or enough vs. Little or not at all)		***	2.497	1.476–4.225
Trust in international health institutions (A lot or enough vs. Little or not at all)			1.255	0.793–1.986
Trust in national political administrators (A lot or enough vs. Little or not at all)		***	2.394	1.569–3.650
Trust in local political administrators (A lot or enough vs. Little or not at all)			0.691	0.470–1.016
Trust in journalists (A lot or enough vs. Little or not at all)		**	1.568	1.042–2.359
	Costant	***	0.018	

Significance: *p < 0.05, **p < 0.01, ***p < 0.001.

**TABLE 4:** Effects on the willingness to be vaccinated as soon as possible: binomial multivariate logistic regression on the data collected in May 2021 (*n* = 977) (Observa Science in Society Monitor, Italy. May 2021).

		Sig.	OR	95% CIs
Gender	Male vs. Female		1.242	0.801–1.924
Age group	15–29 years (ref.)	***		
	30–44 years		0.615	0.320–1.181
	45–59 years		0.676	0.354–1.292
	60 years and over	***	3.910	1.737–8.803
Level of education	Low (ref.)			
	Medium		0.939	0.571–1.543
	High		1.052	0.554–1.998
Main source of information	TV/Radio news (ref.)	***		
	Daily press	**	3.961	1.675–9.371
	Institutional web sources	***	5.359	1.957–14.675
	Social media contacts	***	7.022	2.986–16.513
	Own GP		0.682	0.179–2.601
	Relatives or friends		2.108	0.898–4.949
	Other		2.558	0.778–8.405
	Not looking for information		1.810	0.530–6.173
The most trusted source of information	Indications from national and local institutions (ref.)			
	Own GP		0.636	0.356–1.136
	Newspaper articles		1.187	0.346–4.073
	TV/Radio broadcasts		0.590	0.267–1.306
	Relatives or friends	*	0.334	0.138–0.811
	Web forums or social media contacts		0.897	0.299–2.693
Evaluation of the national scientific experts’ public comm	Opinions to institutions confidentially and not publicly (ref.)			
	Clear and effective interventions		0.345	0.115–1.036
	Too disparate opinions and cause of confusion		0.282	0.111–0.714
	Experts unable to communicate clearly		0.344	0.116–1.020
Trust in physicians and health care workers (A lot or enough vs. Little or not at all)		*	2.326	1.164–4.648
Trust in scientists (A lot or enough vs. Little or not at all)			1.741	0.906–3.346
Trust in national health institutions (A lot or enough vs. Little or not at all)		*	2.046	1.116–3.749
Trust in international health institutions (A lot or enough vs. Little or not at all)			1.484	0.815–2.703
Trust in national political administrators (A lot or enough vs. Little or not at all)			0.859	0.451–1.637
Trust in local political administrators (A lot or enough vs. Little or not at all)		*	2.015	1.145–3.544
Trust in journalists (A lot or enough vs. Little or not at all)			1.305	0.717–2.374
	Costant		0.657	

Significance: *p < 0.05, **p < 0.01, ***p < 0.001.

Results of the binomial multivariate logistic regressions are partially in line with the first hypothesis: Italians who trust scientific experts, healthcare workers, and international health institutions, are more likely to be willing to be vaccinated (H1). Data indicate also that trust in local authorities and national health institutions can also increase the acceptance of anti-COVID-19 vaccines.

Perfectly in accordance with the second hypothesis, Italians who positively evaluate experts’ public communication are more likely to be willing to be vaccinated quickly (H2).

## Discussion

Scientific experts have played a key role during the pandemic in a number of ways: by contributing to the research that has provided insights and responses (including vaccine development), by advising and shaping policy agendas, by communicating extensively across a wide variety of media outlets (TV/radio/daily news, social media). Our data shows that this role and visibility of scientific experts is highly relevant to understand public attitudes towards COVID-19 vaccination. Contrary to a widespread stereotype, specific vaccine hesitancy towards anti-COVID-19 vaccines does not stem or trickle down from generalised scepticism about vaccination and neither from generalised distrust in science. In this pandemic context we are rather observing a “selective scepticism” [30] towards specific aspects of anti-COVID-19 vaccines. The perception of vaccines as being hastily put into production with limited experimentation, the risks of vaccine side effects, the perceived low efficacy in preventing the infection, and the prevalence of political interests over scientific knowledge are just some of the aspects that potentially relate to vaccine hesitant attitudes towards anti-COVID-19 vaccines [31, 32].

Our data also help critically reconsider the role of misinformation or uncareful exposure to informal communication (e.g., the so called “fake news”), which has been very limited in the context of the COVID-19 pandemic. According to data collected by Observa Science in Society Monitor, in all three waves only 4% of Italians believe that social media and web forum are a trustworthy source of information. Indeed, contrary to a widespread concern—often fuelled by media and institutions—social media have played a minor role in information about anti-COVID-19 vaccines and the pandemic in general. Moreover, people who have read or heard about vaccines safety issues on social media then tend to consult their own general practitioner to verify this information [33]. This suggests that Italians are not passive victims of a pervasive infodemic, instead they tend rely on institutional sources of information.

On the other hand, both trust in vaccines’ efficacy and safety and in key actors—health institutions, local authorities, healthcare workers, and scientific experts—are important elements when deciding whether to be vaccinated or not [29, 32, 34]. As our data suggested, trust in scientific experts and perception of the communicative role of experts during the pandemic plays a crucial role in understanding the willingness to get vaccinated. This role articulates its importance in three specific ways. First, in terms of trust: not generalized or abstract trust, but trust in scientists to be able to provide responses to the pandemic threat. Second, in terms of evaluation of the contributions played by experts and the policy institutions they operate within—or collaborate with—to effectively managing the crisis. Finally, and even more interesting, in terms of the communicative performance by experts; the more this is perceived to be clear and helpful, the more it associates with positive attitudes; the more it is perceived as confusing and contradictory, the more it associates with vaccine hesitant attitudes.

We are, of course, aware of the limitations of our study. It will be important to develop and articulate these findings in terms of both future national surveys and international comparisons. However, some relevant implications can be drawn in terms of communication efforts and institutional strategies that are essential to build effective and inclusive vaccination campaigns. It should not be neglected, for example, that most respondents have increasingly perceived, across time, experts’ communication to be a source of confusion. On a global scale, the unprecedented exposure of expert sources across the media has found many institutions unprepared to deal with such responsibility. In many cases, scientific experts’ communication has been guided mostly by personal goodwill and inclination, without enough consideration given to available scholarly knowledge in this area, data on public perception, and audience intelligence (18, 24).

In a period in which public authorities are implementing accelerated approval processes for anti-COVID-19 vaccines, political and health institutions need to adopt effective science communication strategies. Increased investments and efforts should be specifically devoted to developing science communication skills within institutions, sharing data and practices at the international level, conducting comparative studies on the importance how carefully planned science communication strongly relates to mutual trust among experts, citizens and institutions, and more specifically to attitudes towards vaccines.

## Footnotes

1 The Science in Society Monitor, run by non-foundation Observa Science in Society, is the key reference for trends in opinions and attitudes toward science and technology in Italy since 2003.

2 Italian resident population aged over 15 years with a landline phone or registered in the Opinioni.net panel web. Response rates: October 2020, 6.6% (CATI, total number = 4,661) and 39% (CAWI, total number = 1,797); January 2021, 6.7% (CATI, total number = 4,511) and 37% (CAWI, total number = 1,884); May 2021, 10.6% (CATI and CAWI, total number = 9,490).

3 The logistic regression models tested are all multivariate models implemented with the data analysis program SPSS. We performed both binomial and multinomial logistic regression models (with all three categories of the dependent variable) and the results are essentially similar. Therefore, for the sake of parsimony, we have chosen to present binomial logistic regression models and to consider in the models presented only those independent variables that were found significant and/or were commented in the text.

4 This intention has later found strong confirmation by actual vaccination data: in autumn 2021, when most of the vaccinable population had the opportunity to receive the vaccine, the vaccination rate was actually 85%.
